# Suture Techniques for Traumatic Wound Closure in the Emergency Department: A Systematic Review of Cosmetic, Functional, and Infection-Related Outcomes

**DOI:** 10.7759/cureus.87772

**Published:** 2025-07-12

**Authors:** Amr Elfar, Asma Ahmed Osman Mohamed, Ahmed Mahdi, Monzir Adam Ahmed Mohammed, Abdul Mueed Shaikh, Jarallah H. J. Alkhazendar, Ibrahim Adil Hamadelniel Alhadi, Aliaa H Alkhazendar, Ahmed Mohamed, Manahil Awan

**Affiliations:** 1 Emergency Medicine, Aintree University Hospital NHS Foundation Trust, Liverpool, GBR; 2 Surgery, Burjeel Medical City, Abu Dhabi, SAU; 3 General Surgery, Albada General Hospital, Tabuk, SAU; 4 General Surgery, Aqiq General Hospital, Al Aqiq, SAU; 5 Orthopedics, Liaquat National Hospital, Karachi, PAK; 6 General and Emergency Surgery, Lister Hospital, East and North Hertfordshire Teaching NHS Trust, Stevenage, GBR; 7 General Surgery, Royal College of Surgeons of Edinburgh, Edinburgh, GBR; 8 Anatomy, University of Gezira, Wad Madani, SDN; 9 General Surgery, University of Gezira, Wad Madani, SDN; 10 Surgery, Islamic University of Gaza, Gaza, PSE; 11 Orthopedics and Traumatology, Gezira Centre for Orthopaedic Surgery and Traumatology, Wad Madani, SDN; 12 Surgery, Liaquat National Hospital, Karachi, PAK

**Keywords:** cosmetic outcome, emergency medicine, randomized controlled trial, sutures, tissue adhesive, traumatic laceration, wound closure, wound tape

## Abstract

This systematic review aimed to evaluate the comparative effectiveness of alternative wound closure techniques, specifically wound tapes and tissue adhesives, versus traditional suturing in the management of traumatic lacerations in emergency departments. After screening 220 records from databases including PubMed, Cochrane CENTRAL, and Google Scholar, three randomized controlled trials met the inclusion criteria and were analyzed. Across the studies, outcomes assessed included wound closure time, infection and dehiscence rates, and cosmetic or scar-related results. The findings indicate that alternative methods such as wound tape and tissue adhesives offer similar, and in some cases improved, cosmetic outcomes and significantly reduced wound closure times compared to sutures, particularly in low-tension or facial wounds. Infection and dehiscence rates were comparable across interventions. The overall quality of the included studies was satisfactory, with two studies rated as low risk of bias. These findings suggest that in select clinical contexts, particularly where time efficiency and patient comfort are priorities, non-suture methods can be a viable alternative without compromising safety or aesthetic outcomes.

## Introduction and background

Traumatic lacerations are one of the most frequent presentations in emergency departments (EDs), often resulting from blunt or penetrating injuries [1]. Prompt and effective wound closure is essential to achieve hemostasis, prevent infection, restore anatomical integrity, and optimize cosmetic healing. The choice of closure technique directly impacts wound healing outcomes and is therefore a critical component of acute trauma care [2]. Sutures have long been the standard method for closing traumatic wounds, especially in areas with high mechanical stress or deeper tissue involvement. However, recent advancements in wound closure technologies have introduced alternative methods, including tissue adhesives, wound tapes (closure strips), and staples [3]. These techniques offer benefits such as faster application, reduced pain, and the elimination of suture removal, making them attractive options in low-tension wounds or cosmetically sensitive areas like the face.

Multiple randomized controlled trials (RCTs) have assessed the safety and effectiveness of these alternative methods, focusing on outcomes such as infection rates, wound dehiscence, cosmetic appearance, and procedural time. Despite promising results, no consensus has been reached regarding the optimal technique for specific wound types in the dynamic and time-sensitive ED setting. This systematic review evaluates evidence from original RCTs comparing sutures with alternative wound closure methods, aiming to inform clinical decision-making based on infection risk, cosmetic outcomes, and time efficiency.

## Review

Materials and methods

Study Design and Protocol

This systematic review was conducted in accordance with the Preferred Reporting Items for Systematic reviews and Meta-Analyses (PRISMA) 2020 guidelines [4]. The protocol was designed to compare suture techniques and alternative wound closure methods for traumatic lacerations in ED settings, focusing on outcomes related to cosmesis, infection, and procedural efficiency. The review adhered to a clearly defined research framework structured around the PICO model [5]: the Population included patients presenting to the ED with traumatic wounds; the Intervention encompassed various suture techniques and alternative closure methods such as tissue adhesives and wound tapes; the Comparator was conventional suturing; and the Outcomes evaluated included long-term cosmetic appearance, incidence of wound infection or dehiscence, and closure time.

Search Strategy

A comprehensive search of the literature was performed using electronic databases including PubMed, Cochrane CENTRAL, and Google Scholar. The final search was conducted in June 2025. The search strategy employed a combination of Medical Subject Headings (MeSH) and free-text terms such as “wound closure”, “suture techniques”, “traumatic laceration”, “emergency department”, “tissue adhesive”, “wound tape”, “infection”, and “cosmetic outcome”. Boolean operators (AND, OR) were used to refine the search. The search was limited to studies published in English and involving human participants. Additionally, reference lists of selected articles and related systematic reviews were screened manually to identify any relevant studies not captured in the database queries.

Eligibility Criteria

Eligible studies were limited to original RCTs comparing suture-based and alternative wound closure techniques in patients with traumatic lacerations managed in EDs. Included studies had to report at least one of the predefined outcomes: cosmetic appearance, infection or dehiscence rates, and closure time. Studies involving elective surgical wounds, non-traumatic ulcers, animal models, case reports, editorials, or reviews were excluded. Only peer-reviewed, full-text articles published in English were considered.

Study Selection and Data Extraction

The selection process was conducted in two stages. First, titles and abstracts were screened by two independent reviewers to identify potentially relevant studies. In the second stage, full-text articles were retrieved and assessed for eligibility based on the inclusion criteria. Disagreements between reviewers were resolved through discussion and consensus. Data extraction was performed using a standardized form and included study characteristics (author, year, and design), participant details, wound type and location, intervention and comparator descriptions, duration of follow-up, and reported outcomes. Emphasis was placed on extracting numerical data for closure time, cosmetic ratings (e.g., VAS or scar width), and infection or complication rates.

Quality Assessment

The risk of bias for each included RCT was independently assessed using the Cochrane Risk of Bias Tool for Randomized Trials (RoB 2) [6]. This tool evaluates the following domains: the randomization process, deviations from intended interventions, missing outcome data, measurement of outcomes, and selection of reported results.

Results

Study Selection Process

**Figure 1:**
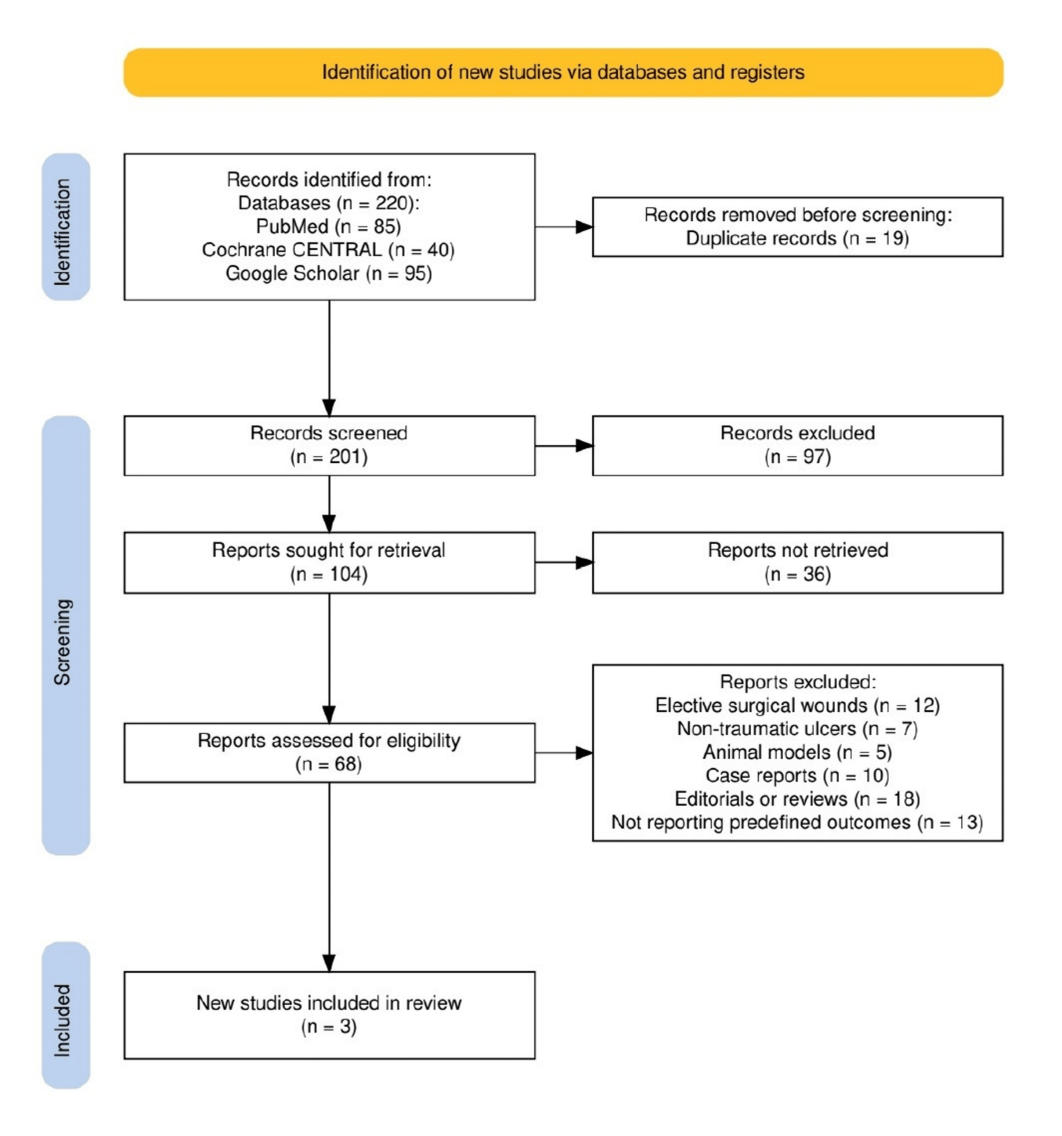
Study selection process in accordance with the PRISMA 2020 guidelines PRISMA, Preferred Reporting Items for Systematic reviews and Meta-Analyses

The study selection process followed the PRISMA 2020 guidelines and is detailed in Figure 1. A total of 220 records were identified through electronic database searches, including PubMed (n = 85), Cochrane CENTRAL (n = 40), and Google Scholar (n = 95). After the removal of 19 duplicate records, 201 unique articles were screened based on titles and abstracts. Of these, 97 were excluded for irrelevance or failing to meet inclusion criteria. Full-text versions of 104 reports were sought, of which 36 could not be retrieved. The remaining 68 articles were assessed in full for eligibility. Ultimately, 65 studies were excluded due to reasons such as being case reports, animal studies, editorials, reviews, or not reporting the predefined outcomes. This left three RCTs that met all inclusion criteria and were included in the final qualitative synthesis.

Characteristics of the Selected Studies

The three RCTs included in this review, summarized in Table 1, were all single-center studies involving patients with traumatic lacerations managed in EDs. Sample sizes ranged from 49 to 124 participants, with wound locations varying from facial injuries to general traumatic lacerations. The interventions assessed included tissue adhesives, wound tapes, and hybrid adhesive-strip systems, each compared against standard suturing techniques. Follow-up durations ranged from 14 days to over three months, depending on the outcome measured. Across the studies, infection and dehiscence rates showed no significant differences between alternative and standard closure methods. One study reported a significantly faster closure time with the adhesive-strip system, while another showed comparable long-term cosmetic results using tissue adhesives. Additionally, wound tape demonstrated improved scar width outcomes in wounds shorter than 20 mm.

**Table 1:** Characteristics of the selected studies ED: emergency department; LS: Leukosan Skin; RCT: randomized controlled trial; VAS: Visual Analogue Scale

Study	Study design	Population (n)	Wound type and location	Intervention	Comparator	Follow-up duration	Primary outcomes	Key results
Kim et al. (2018) [7]	RCT, open-label, single-center	49 patients	Traumatic lacerations, unspecified location, presenting to ED	Leukosan SkinLink (textile strip + tissue adhesive)	Surgical sutures	14 days	Wound closure time, infection, and dehiscence	Closure time significantly shorter with LS (1.48 ± 0.2 seconds vs. 8.8 ± 3.6 min, p < 0.001); no significant difference in infection or dehiscence
Singer et al. (1998) [8]	Prospective RCT, single-center	124 patients (63 adhesive, 61 sutures)	Non-bite, non-crush traumatic lacerations less than six hours old; various locations	2-Octylcyanoacrylate tissue adhesive	Standard wound closure techniques (sutures)	More than 3 months (cosmesis); five to 10 days (infection, dehiscence)	Cosmetic outcome, infection, dehiscence	No significant difference in cosmetic outcome (VAS: 83.8 vs. 82.5 mm, p = 0.72); one infection, two dehiscences (all in adhesive group); not statistically significant
Esmailian et al. (2018) [9]	RCT, single-center	90 patients (45 per group)	Facial traumatic wounds	Wound tape	Standard sutures	Two months	Scar width, wound complications	Overall scar width: no significant difference (2.5 mm vs. 2.9 mm, p = 0.07); for wounds <20 mm, scar width significantly lower with tape (1.7 mm vs. 2.5 mm, p = 0.01); no significant difference in complications

Quality Assessment

The quality assessment of the included studies was conducted using the Cochrane Risk of Bias Tool for Randomized Trials (RoB 2) [6] and is summarized in Table 2. Two of the studies demonstrated a low risk of bias across all evaluated domains, including randomization process, deviations from intended interventions, completeness of outcome data, measurement methods, and selection of reported outcomes. These studies employed clear randomization procedures, maintained complete follow-up, and utilized validated, objective measures for outcomes such as infection and cosmetic appearance. One study was rated as having some concerns, primarily due to an insufficiently detailed randomization process, potential for observer bias in cosmetic scoring, and unclear blinding of outcome assessors. Despite these limitations, all three studies were considered methodologically robust enough to be included in the final synthesis.

**Table 2:** Quality assessment of the included studies VAS: Visual Analogue Scale

Study	Randomization process	Deviations from intended interventions	Missing outcome data	Measurement of outcomes	Selection of reported result	Overall risk of bias	Justification
Kim et al. (2018) [7]	Low risk	Low risk	Low risk	Low risk	Low risk	Low risk	Well-described randomization, complete follow-up, and objective outcomes (time, infection). Open-label, but minimal risk of deviation bias.
Singer et al. (1998) [8]	Low risk	Low risk	Low risk	Low risk	Low risk	Low risk	Proper randomization, valid outcome measures (VAS, photographic grading), minimal attrition, and well-reported results.
Esmailian et al. (2018) [9]	Some concerns	Some concerns	Low risk	Some concerns	Low risk	Some concerns	Randomization process not well-detailed; possible observer bias in cosmetic scoring; outcome assessors not clearly blinded.

Discussion

This systematic review evaluated three original RCTs assessing alternative suture techniques for traumatic wound closure in ED settings. The findings consistently demonstrated that non-traditional closure methods, including tissue adhesives and wound tape, are comparable to standard suturing in terms of infection prevention and cosmetic outcomes, particularly in specific subgroups (Table 3). Kim et al. [7] reported a significantly shorter closure time with Leukosan SkinLink compared to sutures (1.48 ± 0.2 seconds vs. 8.8 ± 3.6 minutes, p < 0.001), with no increase in infection or dehiscence. Singer et al. [8] showed that 2-octylcyanoacrylate yielded cosmetic results statistically equivalent to sutures after 3 months (VAS score: 83.8 vs. 82.5 mm; p = 0.72) and observed no significant difference in infection rates. Esmailian et al. [9] found no overall cosmetic difference between wound tape and sutures (2.5 mm vs. 2.9 mm scar width, p = 0.07), though wound tape showed a statistically superior outcome in wounds less than 20 mm (1.7 mm vs. 2.5 mm, p = 0.01). Importantly, none of the included studies reported a significant increase in wound-related complications with alternative closure methods. These findings support the clinical equivalence or superiority of selective adhesive-based techniques, especially in low-tension or facial wounds, while offering procedural efficiency gains in appropriate settings.

**Table 3:** Comparative analysis of the included studies VAS: Visual Analogue Scale

Study	Intervention vs. comparator	Cosmetic outcome	Infection/dehiscence	Functional efficiency
Kim et al. (2018) [7]	Leukosan SkinLink vs. surgical sutures	Not assessed	No significant difference	Closure time significantly faster (1.48 seconds vs. 8.8 min, p < 0.001)
Singer et al. (1998) [8]	2-octylcyanoacrylate vs. standard sutures	VAS: 83.8 vs. 82.5 mm (p = 0.72); no significant difference	One infection, two dehiscences (in adhesive group); not statistically significant	No significant difference
Esmailian et al. (2018) [9]	Wound tape vs. standard sutures	No significant difference overall (2.5 mm vs. 2.9 mm, p = 0.07); better in <20 mm wounds (1.7 mm vs. 2.5 mm, p = 0.01)	No significant difference in complications	Not reported

Several previous systematic reviews and clinical guidelines, including those from the Cochrane Collaboration and the American Academy of Family Physicians, have examined wound closure techniques in emergency care [10,11]. Consistent with the findings of this review, these sources report that tissue adhesives perform similarly to sutures in low-tension wounds when it comes to cosmetic results and complication rates. Some literature, including a Cochrane review, has noted a slightly higher risk of wound dehiscence with adhesives, although this trend was not statistically significant in the included RCTs. Our review builds on existing evidence by emphasizing that wound tape may offer improved scar appearance in wounds shorter than 20 mm, an observation not widely discussed in earlier studies.

The clinical implications of these findings are important for ED practice. Tissue adhesives and wound tapes appear to deliver comparable outcomes to sutures while offering several procedural benefits [12]. As shown by Kim et al, closure time can be significantly reduced using adhesive systems, which may help alleviate ED overcrowding and improve workflow [7]. In facial lacerations and short wounds, particularly in children or patients with needle aversion, wound tape provides a less invasive and more patient-friendly option [13]. Additionally, since adhesives do not require removal, they can reduce the need for follow-up visits, making them particularly useful in high-volume or resource-limited settings [14].

A key strength of this systematic review is the exclusive inclusion of RCTs, which provides a strong level of evidence. The use of the Cochrane RoB 2 tool allowed for objective quality assessment, and the review focused on outcomes that directly impact clinical decision-making, cosmetic appearance, infection rates, and procedural efficiency. However, limitations must be acknowledged. Only three RCTs met the inclusion criteria, which limits the generalizability of our conclusions. There were also differences in wound types, follow-up durations, and outcome assessment methods, especially for cosmetic evaluation. One study had methodological concerns related to blinding and randomization. Due to these factors, a meta-analysis could not be performed.

Despite promising findings, important gaps in the literature remain. There is a need for larger, multicenter RCTs comparing various closure methods, including sutures, adhesives, staples, and tapes, across different wound types and anatomical locations. Long-term follow-up studies are also needed to evaluate scar formation over time. Pediatric-specific research is particularly important, given unique age-related considerations [15,16]. Furthermore, future studies should incorporate cost-effectiveness analyses and patient-reported outcome measures to better inform practical and patient-centered guidelines. As new closure technologies continue to emerge, ongoing comparative effectiveness research will be essential for optimizing wound care in emergency settings.

## Conclusions

This systematic review highlights that alternative wound closure techniques, specifically tissue adhesives and wound tapes, offer comparable safety and cosmetic outcomes to traditional suturing methods for traumatic lacerations treated in EDs. Notably, these alternatives may provide additional benefits such as reduced procedure time and improved patient comfort, particularly in low-tension or cosmetically sensitive wounds. The review underscores the practical utility of these methods in streamlining emergency care, especially in pediatric or resource-limited settings. By synthesizing evidence from original RCTs, this study contributes valuable insight to the evolving landscape of ED wound management and supports more flexible, patient-centered decision-making.
